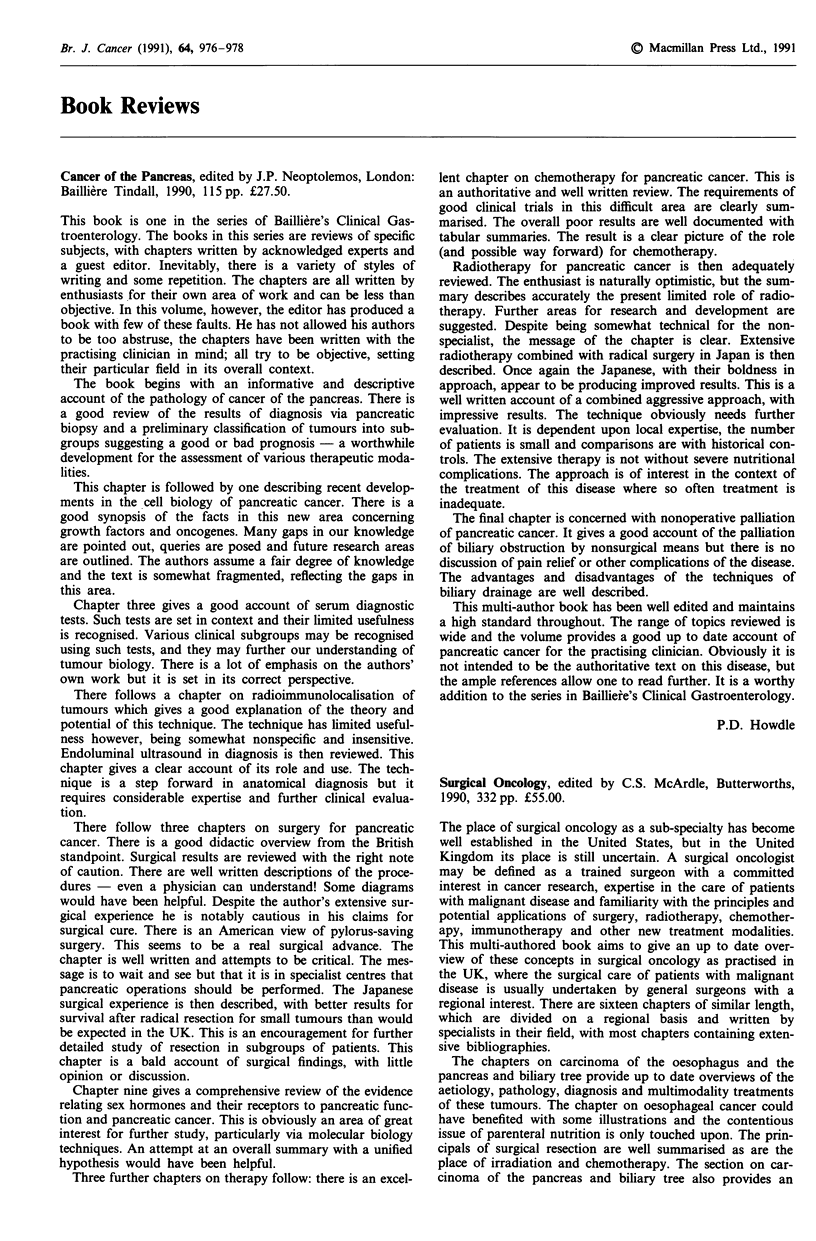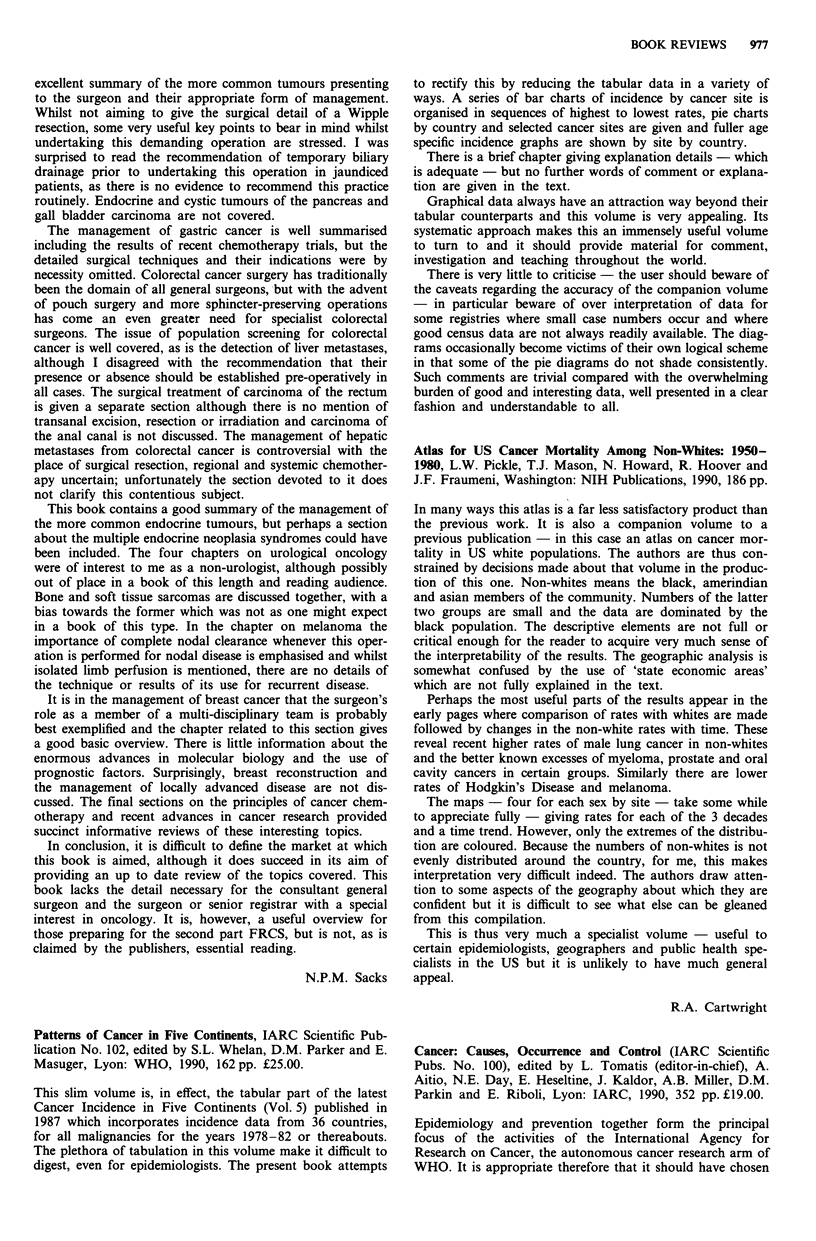# Surgical Oncology

**Published:** 1991-11

**Authors:** N.P.M. Sacks


**Surgical Oncology**, edited by C.S. McArdle, Butterworths, 1990, 332 pp. £55.00.

The place of surgical oncology as a sub-specialty has become well established in the United States, but in the United Kingdom its place is still uncertain. A surgical oncologist may be defined as a trained surgeon with a committed interest in cancer research, expertise in the care of patients with malignant disease and familiarity with the principles and potential applications of surgery, radiotherapy, chemotherapy, immunotherapy and other new treatment modalities. This multi-authored book aims to give an up to date overview of these concepts in surgical oncology as practised in the UK, where the surgical care of patients with malignant disease is usually undertaken by general surgeons with a regional interest. There are sixteen chapters of similar length, which are divided on a regional basis and written by specialists in their field, with most chapters containing extensive bibliographies.

The chapters on carcinoma of the oesophagus and the pancreas and biliary tree provide up to date overviews of the aetiology, pathology, diagnosis and multimodality treatments of these tumours. The chapter on oesophageal cancer could have benefited with some illustrations and the contentious issue of parenteral nutrition is only touched upon. The principals of surgical resection are well summarised as are the place of irradiation and chemotherapy. The section on carcinoma of the pancreas and biliary tree also provides an excellent summary of the more common tumours presenting to the surgeon and their appropriate form of management. Whilst not aiming to give the surgical detail of a Wipple resection, some very useful key points to bear in mind whilst undertaking this demanding operation are stressed. I was surprised to read the recommendation of temporary biliary drainage prior to undertaking this operation in jaundiced patients, as there is no evidence to recommend this practice routinely. Endocrine and cystic tumours of the pancreas and gall bladder carcinoma are not covered.

The management of gastric cancer is well summarised including the results of recent chemotherapy trials, but the detailed surgical techniques and their indications were by necessity omitted. Colorectal cancer surgery has traditionally been the domain of all general surgeons, but with the advent of pouch surgery and more sphincter-preserving operations has come an even greater need for specialist colorectal surgeons. The issue of population screening for colorectal cancer is well covered, as is the detection of liver metastases, although I disagreed with the recommendation that their presence or absence should be established pre-operatively in all cases. The surgical treatment of carcinoma of the rectum is given a separate section although there is no mention of transanal excision, resection or irradiation and carcinoma of the anal canal is not discussed. The management of hepatic metastases from colorectal cancer is controversial with the place of surgical resection, regional and systemic chemotherapy uncertain; unfortunately the section devoted to it does not clarify this contentious subject.

This book contains a good summary of the management of the more common endocrine tumours, but perhaps a section about the multiple endocrine neoplasia syndromes could have been included. The four chapters on urological oncology were of interest to me as a non-urologist, although possibly out of place in a book of this length and reading audience. Bone and soft tissue sarcomas are discussed together, with a bias towards the former which was not as one might expect in a book of this type. In the chapter on melanoma the importance of complete nodal clearance whenever this operation is performed for nodal disease is emphasised and whilst isolated limb perfusion is mentioned, there are no details of the technique or results of its use for recurrent disease.

It is in the management of breast cancer that the surgeon's role as a member of a multi-disciplinary team is probably best exemplified and the chapter related to this section gives a good basic overview. There is little information about the enormous advances in molecular biology and the use of prognostic factors. Surprisingly, breast reconstruction and the management of locally advanced disease are not discussed. The final sections on the principles of cancer chemotherapy and recent advances in cancer research provided succinct informative reviews of these interesting topics.

In conclusion, it is difficult to define the market at which this book is aimed, although it does succeed in its aim of providing an up to date review of the topics covered. This book lacks the detail necessary for the consultant general surgeon and the surgeon or senior registrar with a special interest in oncology. It is, however, a useful overview for those preparing for the second part FRCS, but is not, as is claimed by the publishers, essential reading.

N.P.M. Sacks